# White Matter and Gray Matter Segmentation in 4D Computed Tomography

**DOI:** 10.1038/s41598-017-00239-z

**Published:** 2017-03-09

**Authors:** Rashindra Manniesing, Marcel T. H. Oei, Luuk J. Oostveen, Jaime Melendez, Ewoud J. Smit, Bram Platel, Clara I. Sánchez, Frederick J. A. Meijer, Mathias Prokop, Bram van Ginneken

**Affiliations:** Department of Radiology and Nuclear Medicine, Radboud University Medical Center, Geert Grooteplein 10, 6525 GA Nijmegen, the Netherlands

## Abstract

Modern Computed Tomography (CT) scanners are capable of acquiring contrast dynamics of the whole brain, adding functional to anatomical information. Soft tissue segmentation is important for subsequent applications such as tissue dependent perfusion analysis and automated detection and quantification of cerebral pathology. In this work a method is presented to automatically segment white matter (WM) and gray matter (GM) in contrast- enhanced 4D CT images of the brain. The method starts with intracranial segmentation via atlas registration, followed by a refinement using a geodesic active contour with dominating advection term steered by image gradient information, from a 3D temporal average image optimally weighted according to the exposures of the individual time points of the 4D CT acquisition. Next, three groups of voxel features are extracted: intensity, contextual, and temporal. These are used to segment WM and GM with a support vector machine. Performance was assessed using cross validation in a leave-one-patient-out manner on 22 patients. Dice coefficients were 0.81 ± 0.04 and 0.79 ± 0.05, 95% Hausdorff distances were 3.86 ± 1.43 and 3.07 ± 1.72 mm, for WM and GM, respectively. Thus, WM and GM segmentation is feasible in 4D CT with good accuracy.

## Introduction

Computed Tomography (CT) is increasingly used for diagnosis and treatment planning. Although CT is associated with harmful radiation and limited soft tissue contrast compared to Magnetic Resonance (MR), in many clinical application areas, for example emergency radiology, CT is the preferred modality due to its speed and widespread availability. According to the Organization for Economic Corporation and Development (OECD), the number of CT scanners is approximately twice as large as the number of MR scanners in Europe and almost 1.5 times larger in the United States^1, 2^.

Modern CT scanners are capable of 4D imaging of a whole organ without table movements. Tissue attenuation curves obtained after contrast injection can be used to calculate perfusion values, such as blood flow and blood volume. This allows a combination of anatomical and functional imaging, with large coverage, using a single modality and a single acquisition. We expect that in 5 to 10 years from now scanners with these specifications will be commonly available.

The hallmark of 4D CT is perfusion imaging of the brain to quantify parenchymal hemodynamics, for example in patients suffering from acute ischemic stroke. It has sparked a great interest in the search of quantifying the extent of abnormally perfused brain regions, in particular the tissue at risk (penumbra) which is potentially salvageable by thrombolysis, or by thrombolysis followed by endovascular thrombectomy^3^, and the extent of tissue that already has been permanently damaged (infarct core)^4–6^.

In this work we present a method to segment white matter (WM) and gray matter (GM) in 4D CT images of the brain. Soft tissue segmentation in CT is a research field that has been largely ignored in the past, even though the list of applications is large.

First, WM and GM segmentation enables tissue dependent perfusion analysis. Studies have shown that GM is more vulnerable to ischemia than WM^7, 8^ and this resulted in numerous subsequent studies on determining tissue dependent perfusion thresholds to define core and penumbra^5, 9–11^. Second, segmentation can be used to improve visualization and thus facilitating the detection of early signs of ischemic stroke. In the first hours after ischemic stroke, there is an excessive buildup of edema leading to the loss of WM and GM differentiation on non-enhanced cerebral CT which may appear as loss of the cortical sulci, obscuration of the insular ribbon, midline shift and other appearances^12^. These signs are subtle and sometimes missed in the acute stages^13^. Third, segmentation is a prerequisite for volume measurements. Volume measurements, in particular if normalized with the non-affected contralateral side of the brain, may yield an important diagnostic parameter in the acute phase of ischemia. Brain volume measurements and related measurements such as fractional WM and GM volumes of their combined volume, are important quantitative measures for brain atrophy and have been linked to neurodegenerative diseases. Normal aging is linked to brain atrophy^14^, and total brain volume has been suggested as an integrated measure of health and as a risk predictor for mortality^15^.

Finally, WM and GM segmentation in CT facilitates segmentation of the remaining brain structures, including cerebrospinal fluid (CSF) and cerebral vasculature, and even further and more challenging, automated segmentation and anatomical labeling of the (sub)cortical structures including the brain lobes, giri, thalamic nuclei and caudate from CT. If this can be done robustly, accurately and reproducibly, it would be of great interest for stroke trials, in which specific and functional regions of the brain can be quantitatively taken into account in the analysis.

This list is just partially complete. Other domains of interest are surgical planning, interventional radiology and trauma. However, despite the strong clinical and methodological relevance, related work on WM and GM segmentation in CT is limited and in 4D CT, as we will consider in this work, actually non-existent.

The first method for brain tissue segmentation was presented in 1985 by DeLeo *et al.* ^16^, using intensity thresholding to label CSF, WM and GM from a 2D CT slice. The images in that study originated from the earliest CT scanners that were commercially available, before the invention of slip-ring technology to enable continuous tube rotation, and before the invention of multi-row detectors and spiral scanning techniques^17^. More recent is the work by Gupta *et al.* ^18^ and Kemmling *et al.* ^19^. In Gupta *et al.* ^18^ a heuristic rule-based method with adaptive intensity thresholding was proposed to segment CSF, WM and GM. Development and evaluation of their method was done on the same patient data and their reference standard was manually contoured on high confidence regions only. In Kemmling *et al.* ^19^ a probabilistic atlas was constructed from pre-segmented MR data and was registered to CT to extract CSF, WM and GM, but no quantitative evaluation was provided. Other methods have focused on segmentation of CSF or ventricles only^20–22^.

The objective of this work is to present and quantitatively evaluate an automated method to segment WM and GM in 4D CT.

## Results

### Cross Validation

**Figure 1:**
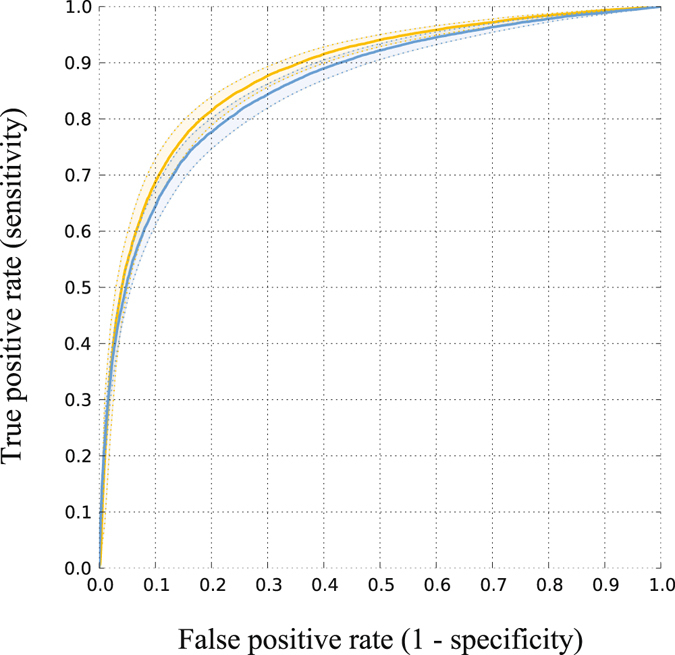
ROC curves of cross validation with the full feature set (yellow) and without the temporal image features (blue). Areas under the curves are 0.88 and 0.86 (*p* < 0.0001), respectively.

In a leave-one-patient-out cross validation the support vector machine (SVM^23^) based method was trained on 21 patients and tested on the remaining patient. The receiver operating characteristics (ROC) curves and their confidence intervals for the cross validations are shown in Fig. 1. The areas under the curves and 95% confidence intervals are 0.88 [0.87, 0.89] for the cross validation with the full feature set, and 0.86 [0.84, 0.87] for the cross validation without temporal image features. Even though the gap is minor, the difference is statistically significant with *p* < 0.0001.

### Quantitative Evaluation

The quantitative results are summarized in Table 1. To put these numbers in perspective we compare them to the performance of MR segmentation algorithms that were compared in an open evaluation framework as part of a challenge^24^. At time of publication, the algorithm that came out ranked first was a multi-feature SVM based method, similarly to ours, using multi-scale intensity and spatial information. Automatic segmentation of WM and GM in MR performs better, particularly in terms of average volume difference (AVD), but in terms of Dice coefficient (DC) and 95% Hausdorff distance (HD), segmentation in CT with our method comes close. The differences between the full feature set and without the temporal features are minimal.

**Table 1:** Quantitative results averaged over all folds of the cross validation, and results from the MR segmentation method ranked first in a challenge^24^.

	*Full feature set*		*Without temporal features*		*First rank MR method*	
	WM	GM	WM	GM	WM	GM
DC [%]	0.81 ± 0.04	0.79 ± 0.05	0.79 ± 0.05	0.78 ± 0.04	88.4 ± 1.2	84.7 ± 1.3
AVD [%]	15.83 ± 10.85	16.48 ± 11.16	14.40 ± 9.22	14.98 ± 9.77	6.0 ± 5.1	6.1 ± 3.3
HD [mm]	12.65 ± 2.18	14.85 ± 3.02	12.78 ± 1.51	14.74 ± 3.10		
MHD [mm]	0.68 ± 0.29	0.58. ± 0.33	0.74 ± 0.26	0.60 ± 0.26		
95% HD [mm]	3.86 ± 1.43	3.07 ± 1.72	4.31 ± 1.10	3.28 ± 1.49	2.4 ± 0.5	1.9 ± 0.4
CMD [mm]	1.35 ± 0.26	0.74 ± 0.19	1.50 ± 0.20	0.80 ± 0.17		

### Qualitative Evaluation

**Figure 2:**
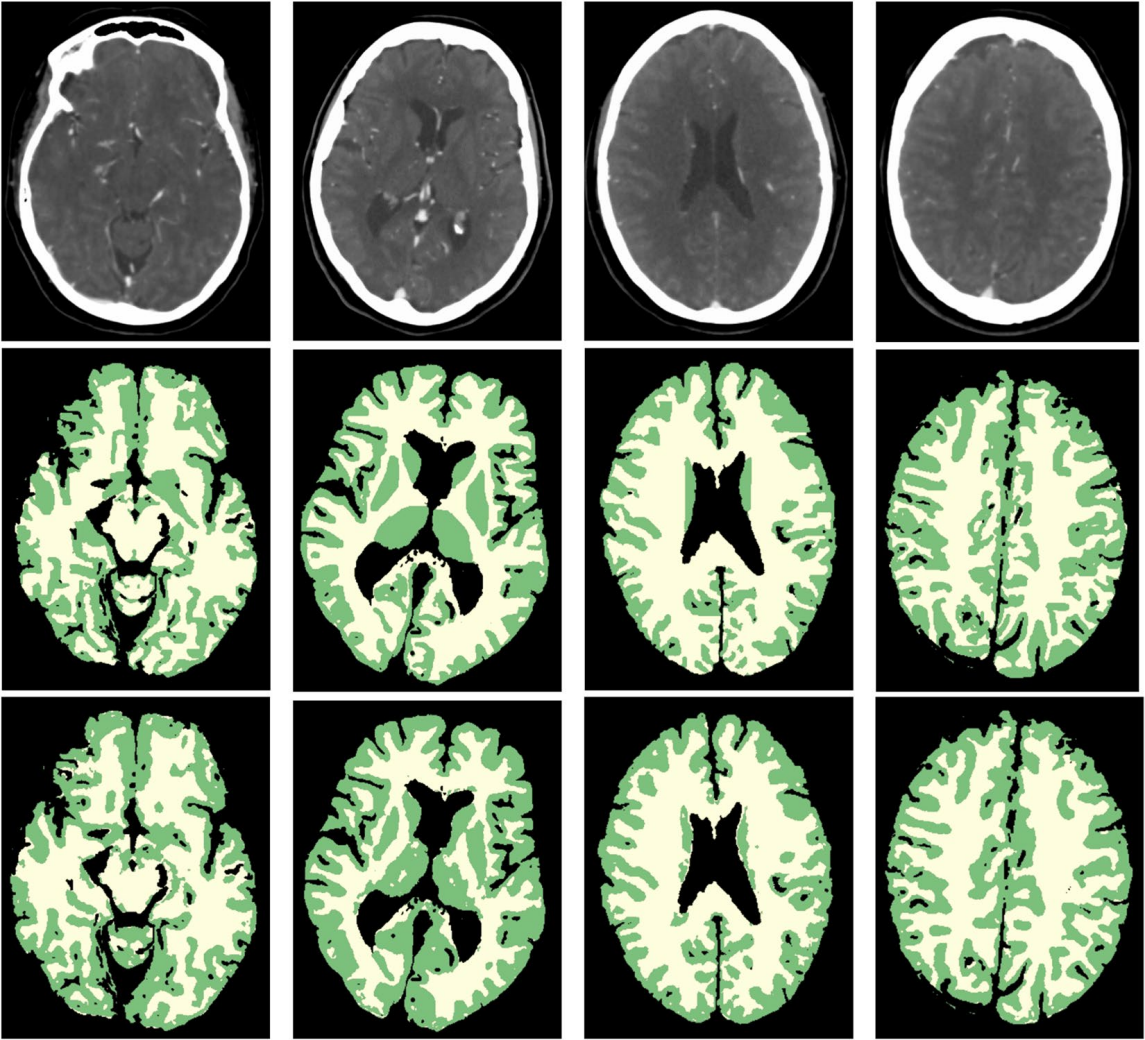
WM and GM segmentations of four different patients. From left to right: axial slices at approximately the level of the Circle of Willis, basal ganglia, lateral ventricles and centrum semiovale. From top to bottom: weighted temporal average (window width/level 300/100 HU); the reference standard; the final WM (yellow) and GM (green) segmentation with the proposed method.

**Figure 3:**
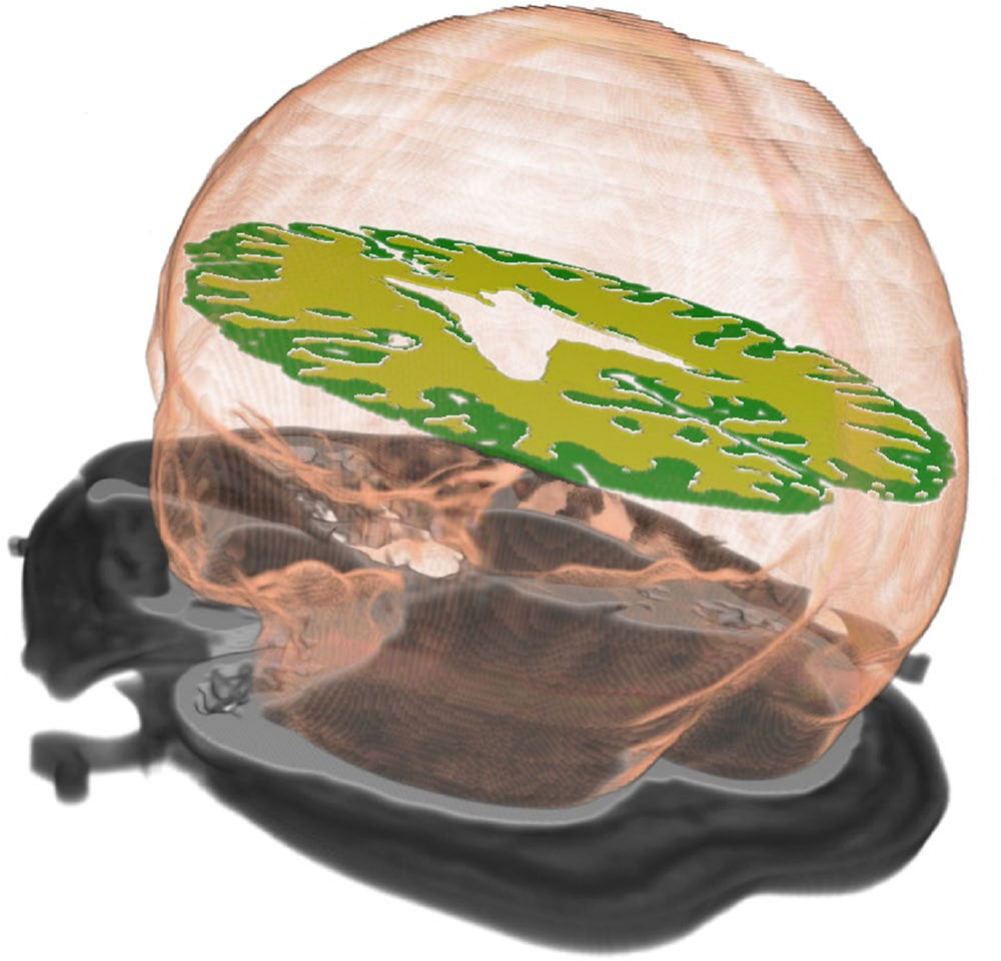
Volume rendering of the intracranial, WM and GM segmentations in 4D CT.

Representative slices of four patients are shown in Fig. 2. An example volume rendering is shown in Fig. 3. Overall, the segmentation results were evaluated as good, with the separation of WM and GM at the cortex good to excellent. GM segmentations at the cortex generally had less variations in thickness compared to the reference standard. Segmentations at the level of the Circle of Willis proved to be more difficult than at higher slice levels of the brain, particularly compared with segmentations at the level of the centrum semiovale, because of the presence of the large-varying arterial vasculature and the anterior lobe. However, this was the case for the reference standard as well. The basal ganglia segmentations were good but in some cases the boundaries had more irregularities than the corresponding reference standard, suggesting that an additional postprocessing step to smoothen the boundaries may improve the segmentations. Of note, the basal ganglia were all manually corrected and refined by the expert observer, and were often poorly visible in the 4D CT image. Segmentation of the caudate nucleus was evaluated as acceptable to good and again, a smoothening step or closed-form boundary evolution, e.g. using a levelset or simplex mesh, with curvature constraints may improve the final segmentations. At the level of the centrum semiovale, the WM and GM segmentations were evaluated from good to excellent, and often perceived closer to the real anatomical underlying structures as shown on the weighted temporal average (WTA) than the corresponding reference standard.

## Discussion

The main contribution of this work is that we have shown the feasibility of segmenting white matter and gray matter in 4D CT.

The densities of WM and GM are very close. A conventional CT acquisition at standard dose is therefore not the most suitable modality for soft tissue differentiation. However, the strong progress of CT scanner technology currently brings soft tissue segmentation in the realm of CT imaging. This work is a proof of that statement. We foresee a range of clinical applications, including tissue dependent perfusion analysis and automated detection and quantification of cerebral related pathology. As part of the overall methodology, we have shown how to optimally construct a 3D temporal average image from 4D CT weighted according to the exposures of the individual time points of the 4D CT protocol.

This WTA image is a key image in our methodology. It is both used for intracranial segmentation and features extraction. In this work it was used to coarsely segment the outer skull, to build scale spaces of zeroth and first order Gaussian derivatives, for local contrast normalization and to coarsely segment CSF and the cerebral vasculature. The WTA image is the highest quality 3D image if only quantum noise is considered. Importantly, it is independent of the distribution of the total exposure over the different acquisitions; it is only influenced by the total exposure. In other words, the WTA has the same noise levels as if it was acquired using one volumetric scan with an exposure equal to the total exposure of the 4D acquisition. This may be an important finding in the design of 4D CT protocols. It also implies that our comparison study on segmentation accuracies with the full feature set and without temporal features can be considered as the minimally achievable gap between the ROC curves. A comparison between a 4D CT acquisition and a conventional CT or CTA at standard exposures most likely widens this gap.

In addition, a method for intracranial segmentation in CT has been described, which is based on atlas registration and subsequent levelset evolution. Until now, this problem was only considered in MR, presumably because in CT intensity thresholding followed by basic image processing usually suffices to obtain acceptable results for many applications. The atlas- and levelset-based method is preferred because it allows for a finer control of defining the boundaries between the intracranial space and skull, particularly at the level of the Circle of Willis, and is more robust to e.g. the presence of imaging artifacts.

Note that even though the proposed methods for intracranial, vessel and CSF segmentation make use of the WTA because it has the lowest image noise, these steps can be improved by considering other features and methodology. For example, an important feature for vessel segmentation is the area under the curve. Accurate and robust segmentation of these anatomical regions in 4D CT is part of our current work.

The intra-patient registration step gave good results in all patients. This registration step is routinely used to process our research archives including hundreds of 4D CT imaging data and only in rare cases, often already marked as non-diagnostic, produces results unsuitable for further interpretation or processing. Inspection of the temporal difference images after registration reveals that errors occur mainly in the frontal area around the nose and jaw. Parenchymal tissue is unaffected. Therefore, the impact of potential registration errors on the WTA image is minimal. Weighted averaging increases image contrast of WM and GM; for segmentation of the cerebral vasculature however, particularly distal in the vessel tree containing many partial volume voxels, a further refinement of the intra-patient registration may be required to achieve sub-voxel delineation of the vessel boundaries.

Quantitative analyses showed good performance of the method. A Dice coefficient of approximately 0.80 was obtained and the 95% Hausdorff distances were 3.9 and 3.1 mm for WM and GM respectively. In terms of these measures, our method comes close compared to state-of-the-art MR segmentation methods, which is a promising results considering the fact that developments in MR already spans several decades. However, the average volume difference was approximately 15% compared to the reference standard. This is quite large, and the main reason is that our method has difficulties in segmenting the complete basal ganglia. Voxel classification (alone) may not be the best approach for segmenting these structures and incorporating model information will likely improve the results.

The qualitative evaluation showed good results. At the level of the centrum semiovale, the segmentation results of our method were actually perceived closer to the underlying anatomical structure than the reference standard.

The reference standard was provided by MR. Not every patient had a follow-up MR as this is not part of standard clinical care. The different resolutions of the images (the MR image had voxel sizes of 5.5 mm in *z*-direction, while the CT had 0.5 mm) is a limitation. We have chosen not to downsample the CT image - thereby reducing noise, but to upsample the MR image instead, to be able to make a fair assessment of the performance of the method on the full-resolution 4D CT as is used in clinical practice. Another limitation is that the quality of the reference standard is limited by the quality of the MR segmentations. The approach taken in this work was using FSL for the initial segmentation followed by manual refinements on selected slices. These slices were agreed upon with the clinical experts and were located in the supratentorial brain. Registration of the MR image, FSL segmentation and finally the manual refinement is a costly and time consuming procedure and will not necessarily leads to the highest quality reference standard. Also, this manual procedure limits a subset of the contextual features to slice-based distances only, instead of fully exploiting the 3D spatial information. Because the WTA image showed acceptable WM and GM contrast, we are currently working on creating a reference standard by directly annotating the WTA image in 3D with the dedicated tools.

Next to these improvements of the reference standard, our next steps are the assessment of the observer variability, further development of methodology to increase its accuracy and robustness, and expanding towards the infratentorial brain and other applications, including the detection and segmentation of cerebral pathology.

## Methods

The method consists of four steps: atlas based intracranial segmentation, coarse CSF and vessel segmentation, WM and GM feature extraction, and voxel classification.

A key image throughout our methodology is the 4D image averaged over time. Because the CT protocol contains multiple acquisitions at different exposures, we first construct a weighted average to maximize the signal to noise ratio. We call the resulting image the weighted temporal average (WTA).

### Weighted Temporal Average

There are three sources of noise in CT imaging: quantum noise, electronic noise and structural noise. Quantum noise is caused by the Poisson distributed fluctuations of the X-ray photons in the X-ray beam. Electronic noise is caused by the electronic read out of the detector elements. Structural noise is caused by the spatially fixed variation of the gain leading to e.g. ring artifacts. In normal clinical routine quantum noise is the predominating noise factor. It is inversely proportional to the square root of the exposure.

Consider a 4D CT protocol consisting of *N* volumetric acquisitions with each image *i* having exposure *E*_*i*_ and noise *σ*_*i*_, then the total variance after temporal averaging is given by:

1 $${\sigma }_{WTA}^{2}=\sum _{i=1}^{N}{\omega }_{i}^{2}{\sigma }_{i}^{2}$$

with *ω*_*i*_ the weighting factor of image *i*, subject to ∑*ω*_*i*_ = 1. We use the method of Lagrange to minimize Equation 1.

2 $$ {\mathcal L} (\omega ,\sigma ,\lambda )=\,\sum _{i=1}^{N}{\omega }_{i}^{2}{\sigma }_{i}^{2}+\,\lambda (\sum _{i=1}^{N}{\omega }_{i}-1)$$

Taking the partial derivative to *ω*_*i*_ and set to zero to obtain a minimum:

3 $$\frac{\partial  {\mathcal L} (\omega ,\sigma ,\lambda )}{\partial {\omega }_{i}}=2{\omega }_{i}{\sigma }_{i}^{2}+\lambda =0$$

Setting the sum of all partial derivatives to zero:

4 $$\sum _{i=1}^{N}\frac{\partial  {\mathcal L} (\omega ,\sigma ,\lambda )}{\partial {\omega }_{i}}=\sum _{i=1}^{N}2{\omega }_{i}{\sigma }_{i}^{2}+N\lambda =0$$

Substituting λ in Equation 4 for the term given in Equation 3:

5 $${\omega }_{i}{\sigma }_{i}^{2}=\sum _{i=1}^{N}\frac{{\omega }_{i}{\sigma }_{i}^{2}}{N}$$

Observing that the term $\sum {\omega }_{i}{\sigma }_{i}^{2}/N$ is a constant:

6 $${\omega }_{i}=\frac{k}{{\sigma }_{i}^{2}}$$

Because ∑*ω*_*i*_ = 1:

7 $$k=\frac{1}{{\sum }_{i=1}^{N}1/{\sigma }_{i}^{2}}$$

Combining Equations 6 and 7 gives the solution:

8 $${\omega }_{i}=\frac{1/{\sigma }_{i}^{2}}{{\sum }_{i=1}^{N}1/{\sigma }_{i}^{2}}$$

In other words, to obtain the minimal noise in the averaged image, the weighting factor of each image should be proportional to the reciprocal variance. For exposure *E*, neglecting structural noise, the following relation holds:

9 $$\sigma \propto \frac{1}{\sqrt{E}}$$

Combining Equations 8 and 9:

10 $${\omega }_{i}=\frac{{E}_{i}}{{\sum }_{i=1}^{N}{E}_{i}}$$

**Figure 4:**
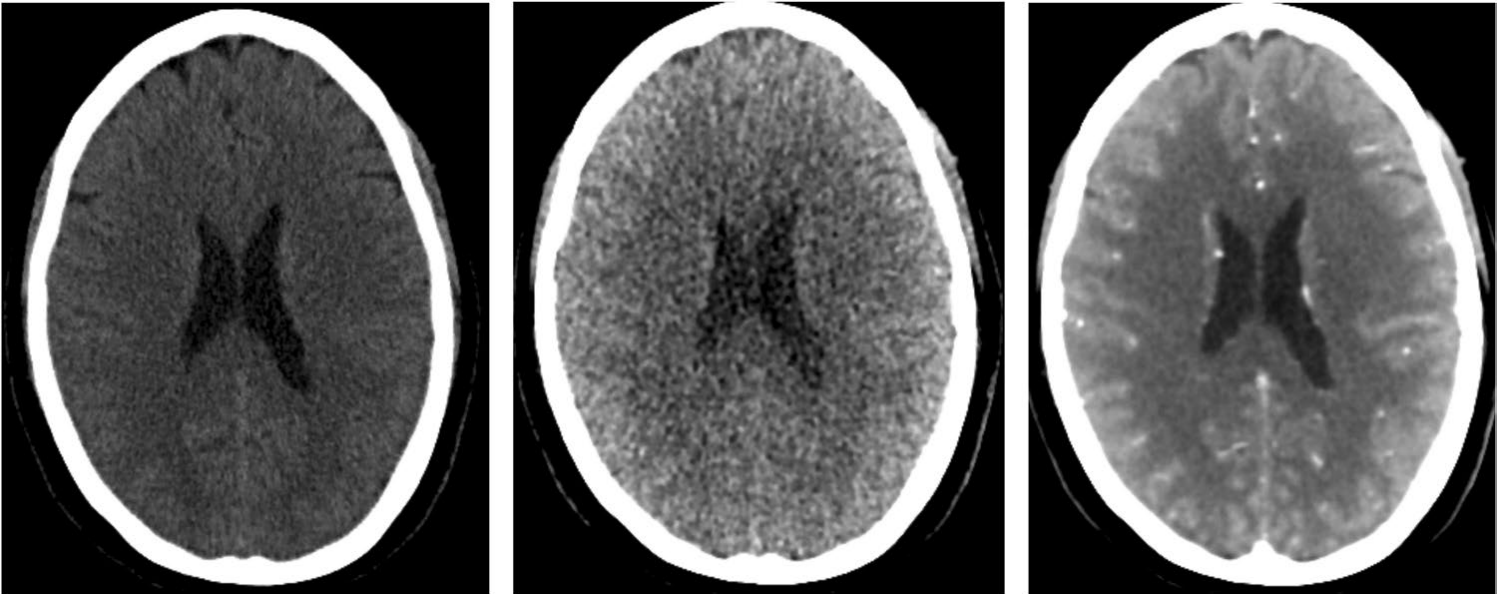
Axial slice at the level of the ventricles of a non-contrast CT acquired with 220 mAs exposure (left), the first time point of the 4D CT at 200 mAs, and the weighted temporal average of the 4D CT at a total exposure of 2250 mAs. All images are visualized with the same window level and width of 60 and 120 HU.

This implies that the total noise in the exposure-weighted temporal average image is independent of the distribution of the total exposure over the different acquisitions. It is only influenced by the total exposure. The WTA image is the highest quality reconstructed 3D image if only quantum noise is considered (Fig. 4), and is therefore use in the subsequent steps. The total noise in the WTA image results from combining Equations 1 and 5:

11 $${\sigma }_{WTA}=\sqrt{\frac{1}{{\sum }_{i=1}^{N}1/{\sigma }_{i}}}$$

### Intracranial Segmentation

Intracranial segmentation was carried out on the WTA images and propagated to the remaining time points. One patient was used as atlas image, in which soft tissue was semi-automatically segmented. Semi-automatic segmentation was obtained by region growing and manually corrected with ITK-SNAP (version 3.2.0)^25^. The soft tissue was slightly under segmented, which is not critical because it is used as initialization of a subsequent levelset for the final segmentation.

Inter-patient registration was carried out on bone obtained by intensity thresholding at 200 HU followed by selection of the largest connected component. The open-source framework Elastix (version 4.400)^26^ was used with the following parameters: affine transformation, adaptive stochastic gradient descent, mean of square differences for the cost function, multi-scale using recursive Gaussian pyramid, i.e. smoothing and down sampling, at 16, 8, 4, 2, and 1 voxel scales, 32 histogram bins, and maximum 500 iterations. The registered intracranial atlas was further refined by application of a geodesic active contour (GAC) with curvature and advection constraints^27^ steered by a gradient based speed function. The partial differential equation governing the GAC is given by:

12 $${{\rm{\Phi }}}_{t}+g\cdot (1+\varepsilon \kappa )|\nabla {\rm{\Phi }}|+\alpha \nabla g\cdot \nabla {\rm{\Phi }}=0$$

With Φ the levelset function, curvature weighting $\,{\epsilon }=1.0$, mean curvature *κ*, advection weighting *α* = 5.0, and *g* the gradient based speed function:

13 $$g(I)={e}^{-{(\frac{\Vert \nabla I\Vert }{\gamma })}^{2}}$$

**Figure 5:**
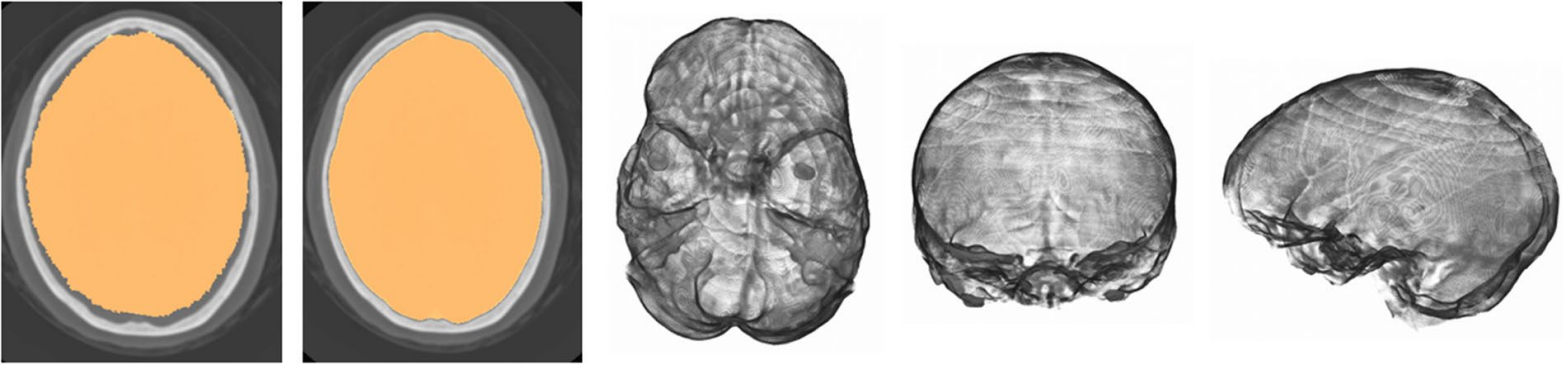
The result of intracranial segmentation after atlas registration (first panel) and refined segmentation after levelset evolution (second panel). Corresponding volume renderings (axial view from the skull base, coronal and sagittal view).

With ∇*I* the image gradient calculated using Gaussian derivative at scale 1.0 voxels and gradient weighting factor *γ* = 80. The intracranial mask obtained from atlas registration was eroded with a structuring element with 3 voxels radius and a distance transform was applied to obtain the initial levelset. The levelset ran with a maximum of 250 iterations, had maximum root mean square error of 0.001 as stopping criterion and used a narrow band implementation only updating the levelset within a 9 voxel layer during iterations. An example of intracranial segmentation before and after the levelset refinement is shown in Fig. 5.

### Coarse CSF and Vessel Segmentation

**Figure 6:**
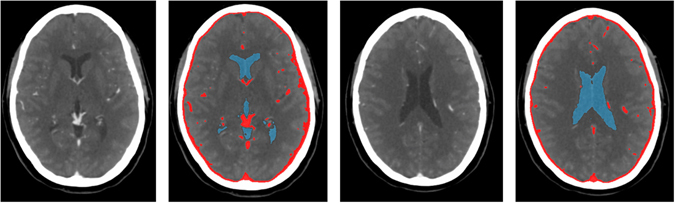
Examples of CSF and vessel segmentation using intensity information and morphological operations in the WTA image (window level/width 100/300 HU). These structures are masked in the intracranial image before WM and GM segmentation.

Next CSF and vessels were coarsely segmented in the WTA image and masked out from the intracranial image. A slight over segmentation of CSF and vessel is allowed to ensure that only soft tissue remains to be segmented. To this end the mean and standard deviation were determined of the intensity histogram within [0,400] HU. An upper intensity threshold *μ* − 1.96*σ* was applied to segment CSF and a lower intensity threshold *μ* + 1.96*σ* to segment the vessels, followed by erosion with a structuring element with 1 voxel radius to remove potential false positive voxels due to noise, and connected region growing using the same thresholds. The CSF mask was subjected to a morphological closing operation with a structuring element with 1 voxel radius. Examples of the CSF and vessel segmentations are shown in Fig. 6.

### WM and GM Feature Extraction

The remaining voxels after intracranial, CSF and vessel segmentation, are representing WM and GM. To segment these we used a pattern recognition framework consisting of feature extraction and voxel classification. Thus it is formulated as a two-class classification problem and we adopted the convention that WM represented background and GM foreground. Per voxel, the following types of features were extracted: intensity-based, contextual-based, and temporal-based features. Scale and parameter values were found by pilot experiments and visual inspection of the feature images. Settings that led to increased WM and GM contrast were selected.

The intensity-based features consisted of (between parentheses the total number is given): the WTA image and its scale-space representation by convolution with a Gaussian function at scales 1.0, 2.0 and 4.0 mm (4). The gradient magnitude of the WTA image by convolution with a Gaussian derivative at scales 1.0, 2.0 and 3.0 mm (3). Locally intensity histogram equalized WTA images with the following parameters *α* = 0.3, *β* = 0.5 and window radii of 5, 10 and 15 voxels^28^ (3).

The contextual-based features consisted of: the slice-based distance to the skull, to the CSF, and to the vessels, normalized by the maximum distance on the slice (3). The skull was coarsely segmented by lower thresholding the WTA image at 200 HU followed by selecting the largest connected component. The cumulative position within the intracranial mask normalized by its total volume in the x, y and z direction, which indicates per voxel which percentage of volume is next, above or behind it ref. 29 (3).

The temporal-based features consisted of: the first 14 time points of the 4D CT acquisition (only this subset because then the number of features are the same for all patients without requiring extrapolation), and its scale-space representation obtained by spatial Gaussian blur at scales 1.0, 2.0 and 4.0 mm, each time point taken as an individual feature (56). A temporal scale-space was constructed obtained by temporal Gaussian blur at scales 1.0, 2.0 and 4.0 seconds and each time point taken as an individual feature (42). The area under the curve of the temporal gradient magnitude of the first 14 time points of the 4D CT^30^, with temporal gradient calculated by convolution with a Gaussian derivative at scales 1.0, 2.0, 4.0, 6.0 and 8.0 seconds and the area under the curve numerically approximated using the trapezoidal rule (5). And finally the time to the first peak of the 4D CT (1). For all tissue curves, time information was encoded in absolute seconds relative to the first acquisition which was set at 0 seconds.

Thus per voxel a feature vector with 10 + 6 + 104 = 120 elements was constructed. All features were normalized to zero mean and unit standard deviation.

### Voxel Classification

A non-linear SVM with Gaussian radial basis kernel function

14 $$K({{\boldsymbol{x}}}_{{\boldsymbol{i}}},{{\boldsymbol{x}}}_{{\boldsymbol{j}}})={e}^{-\gamma \Vert {{\boldsymbol{x}}}_{{\boldsymbol{i}}}-{{\boldsymbol{x}}}_{{\boldsymbol{j}}}\Vert }$$

was used for the two-class classification problem^23^. Pilot experiments with other statistical classifiers were performed including k-nearest neighbor, GentleBoost and linear discriminant analysis, and Gaussian radial basis SVM showed the best performance for this task. Probability estimates were obtained by first fitting a sigmoid function to the decision values to get the pairwise class probabilities^31^, followed by solving the optimization problem as formulated in Wu *et al.* ^32^. The library LIBSVM (version 2.89)^33^ was used in the implementation.

### Parameter Optimization

The parameter settings for intracranial, CSF and vessel segmentation were found by pilot experiments. The parameter settings for WM and GM segmentation were found by a quantitative cross validation in a leave-one-patient-out manner. At a sampling rate of 1.0%, the average number of samples per fold was 51.4 k for training and 2.4 k for testing. But even at this rate the training phase of SVM is slow, and cross validation can easily become computational intractable. Ideally, for each fold multiple kernels and parameters should be explored and optimized. But the training sets in each fold are very similar, and there are many similar training samples for the two classes, we fix the kernel for all folds and single out two parameters before cross validation: the penalty parameter *C* of the error term and the parameter *γ* of the Gaussian kernel. These two parameters are most important in a Gaussian radial basis SVM^33^. The parameters were optimized in one training fold using a full exhaustive grid search in the range *C* ∈ [2^4^, …, 2^15^] and *γ* ∈ [2^−16^, …, 2^−5^], resulting in 144 training stages, optimizing the area under the ROC curve *A*_*z*_. The search resulted in optimal values *C* = 2^10^ and *γ* = 2^−11^. These values were kept constant in the remaining folds.

### Cross Validation

In a leave-one-patient-out cross validation the SVM classifier was trained on 21 patients and tested on the remaining patient. To quantitatively assess the added value of temporal information we performed the same cross validation experiment without the group of temporal features. Each fold generated a trained SVM classifier and a soft classification of the test image, indicating per voxel the probability it belongs to either WM or GM. Because the same SVM classifier was used for each fold and because the training and test sets are representative samples of the total population, we merged the resulting soft classification of each fold and then generated the ROC curve representing the overall performance of cross validation. To test for significance of the difference between the two approaches we used bootstrapping^34^. Random sampling with replacement was carried out for each approach, with 50,000 samples and *A*_*z*_ values with 95% confidence intervals were reported.

### Quantitative Evaluation

Per fold, a hard classification, i.e. a binary segmentation of WM and GM, of the test set was obtained by thresholding the soft classification at 0.5. These segmentations were then compared to the reference standard using the following measures: Dice coefficient (DC), Hausdorff distance (HD), modified HD (MHD), 95% percentile HD, contour mean distance (CMD) and absolute volume difference (AVD). The HD measures follow the definitions as given in Dubuisson *et al.* ^35^. The CMD is the mean distance between the segmentations along their boundary voxels. Because for each fold the test set consisted of non-consecutive slices, these measures were first calculated per slice, then their mean (DC, CMD, AVD) or maximum (HD, MHD, 95% HD) were taken for all slices. Finally, the mean and standard deviation per evaluation measure were taken over all folds and reported.

### Qualitative Evaluation

The binary segmentations of the cross validation were visually inspected by one resident in radiology (MTHO) with 5 years of experience with stroke imaging.

## Materials

### Patient Inclusion

The ethics committee of our institution waived informed consent for all patients in this retrospective study. We searched our database for patients who received both a 4D CT and MR scan. Patients were scanned on suspected stroke or arteriovenous malformations (AVMs) at the Radboud University Medical Center, Nijmegen, The Netherlands. Most patients received the MR on the same day or only a few days after CT. All data were visually inspected for image quality. Exclusion criteria were the presence of metal or severe motion artifacts, major differences between CT and MR e.g. due to changing pathology, or failure of registration or segmentation of the MR images. The latter failures were due to the presence of coils and in one occasion the presence of a drainage tube. In total 22 patients (8 male, 14 female, median age 63 years, range 31 to 89 years, *n* = 20 stroke, *n* = 2 AVM) were included. Acquisition dates ranged from October 2011 to August 2013. Inspection of the images and patient reports revealed that there were no large perfusion deficits in the CT perfusion images nor large affected regions in the corresponding MR.

### Acquisition Protocol

CT imaging was performed on a 320-row CT scanner (Toshiba Aquilion ONE, Toshiba Medical Systems Corporation, Japan). The 4D CT protocol for the patients with suspected stroke started 5 seconds after contrast injection with a high dose volumetric scan at 200 mAs exposure, followed after 4 seconds by 13 scans every 2 seconds at 100 mAs, followed by 5 or 10 scans every 5 seconds at 75 mAs depending on if the late contrast phase-out needed to be visualized as well. Thus in total 19 or 24 volumetric acquisitions were made. The 4D CT protocol for the patients with suspected AVMs included the first 14 volumetric acquisitions at the same time points and exposure settings as the stroke protocol. Each volumetric scan had 16 cm coverage and was made at 80 kV at 0.5 seconds rotation time. All reconstructions were made with a smooth convolution kernel (FC41) and the reconstructed images had 512 × 512 × 320 voxels with voxel sizes of 0.47 × 0.47 × 0.5 mm.

Twenty patients received a MR scan on a 1.5 Tesla scanner and 2 patients on a 3.0 Tesla scanner (Magneto Avanto and Magnetom Trio Tim, Siemens Erlangen, Germany). The T1w images were scanned with the following parameters in the range: TE = 3.0 … 17 milliseconds, TR = 450 … 2000 ms, flip angle = 15 … 90 degrees, voxel sizes of approximately 0.6 × 0.6 × 5.5 mm and image sizes of approximately 384 × 318 × 26 voxels. Thirteen patients received contrast agent prior to scanning for visualization of the cerebral vasculature.

### 4D CT Registration

The volumes of the 4D CT protocol were rigidly registered to the first time point to correct for possible patient movements. Intra-patient registration was carried out on the bone structures only, obtained by intensity thresholding at 600 HU followed by selection of the largest connected component. A higher threshold and therefore a smaller mask was sufficient compared to the inter-patient registration because the potential misalignment is smaller. Elastix was used with the following parameters: Euler transform (rotation and translation), advanced mean squares, 32 histogram bins, maximum number of iterations 500, automatic initialization using center of gravity, standard gradient descent for minimization with *a* = 0.001, *A* = 50 and *α* = 0.60, and a multi-scale approach using Gaussian smoothing and down-sampling at 4 scales of 8, 4, 2 and 1 voxels.

### Reference Standard

The MR image was used to obtain a reference standard. To this end, the MR image was registered to the WTA using an affine registration and the result was subsequently refined by a nonrigid B-spline registration. The nonrigid registration was required because the MR images were much smaller than the CT images in *z*-direction. Also there were several days between the acquisitions and minor differences of parenchyma is possible, particularly if the CT has been acquired in acute stroke, which is pathophysiologically a highly dynamic situation^36^. The affine registration had the following parameters: advanced Mattes mutual information, 32 histogram bins, maximum number of iterations 500, automatic initialization using center of gravity, adaptive stochastic gradient descent for minimization, and a multi-scale approach using Gaussian smoothing at 4 scales of 8, 4, 2 and 1 voxels. The nonrigid B-spline registration was applied to soft tissue between intensities of 200 HU and 400 HU in the WTA image, and had the following parameters: advanced Mattes mutual information, 32 histogram bins, maximum number of iterations 2000, standard gradient descent with *a* = 10000, *A* = 100 and *α* = 0.6, order 3 for the final B-spline interpolation, and no multi-scale approach.

The registered MR image was then analyzed with FSL^37^. BET^38^ and FAST^39^ were used of this library for brain and soft tissue segmentation, respectively. BET ran with fractional intensity threshold at *f* = 0.45 and vertical gradient at *g* = 0.0 We tried successively and mutual exclusively the following BET options until a good brain segmentation was obtained: reduction of image bias and residual neck voxels (B), cleanup of residual eye and optic nerve voxels (S) and robust brain center estimation (R). The resulting brain image was then fed into FAST which ran with the following parameters: number of tissue types *n* = 3 (WM, GM and CSF), spatial smoothness *H* = 0.35, number of iterations for bias field removal *I* = 4, bias field smoothing *l* = 20 and the use of a prior probability map for initialization (a). The number of tissue types was also set at *n* = 3 for the MR images with contrast, as experiments did not show improvements in WM, GM, and CSF segmentation if a fourth class was included for the vessel voxels.

**Figure 7:**
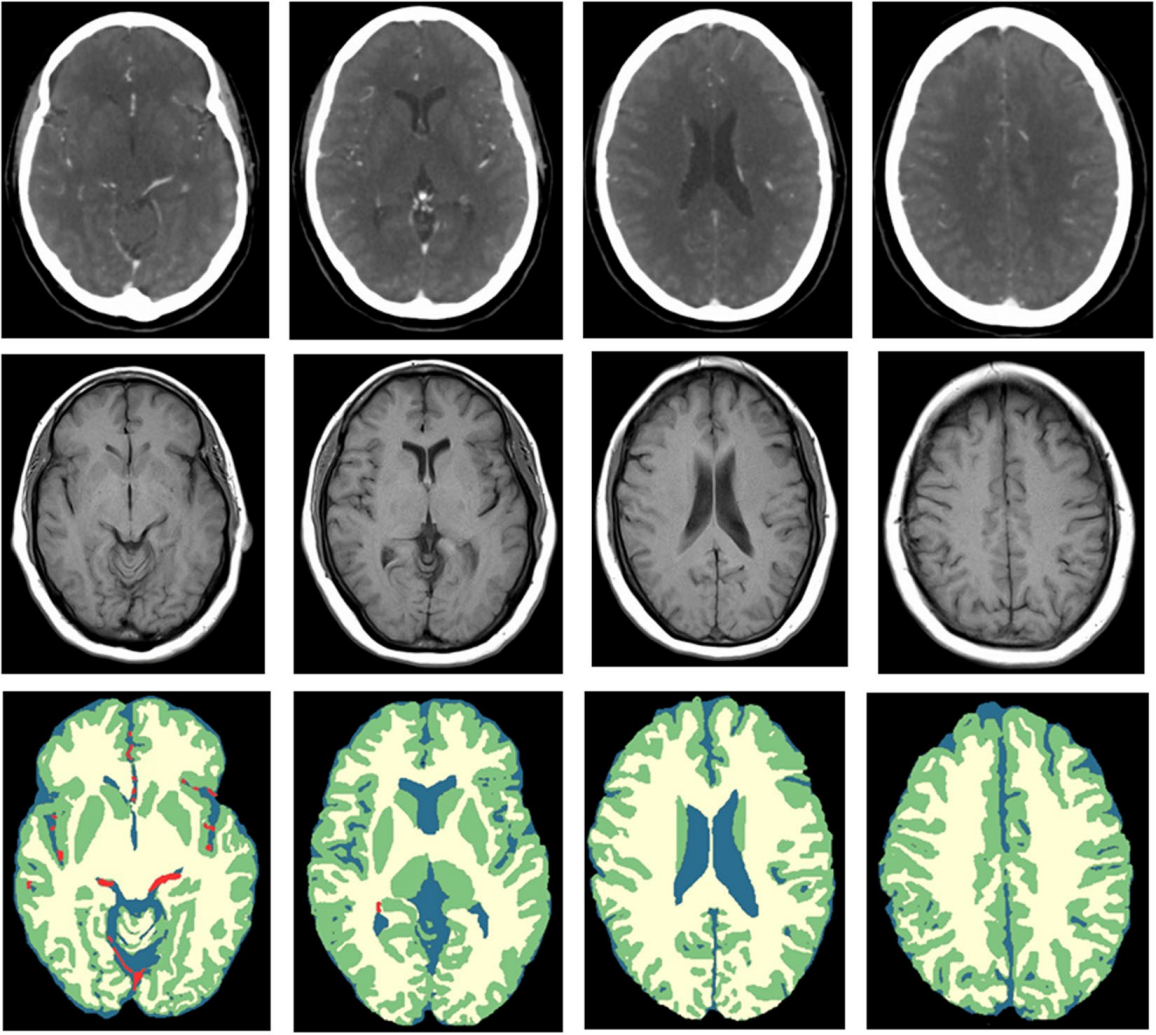
Axial slices at approximately the level of the Circle of Willis, basal ganglia, lateral ventricles and centrum semiovale (from left to right). From top to bottom: WTA (window width/level 300/100 HU); the registered MR T1w image; the final segmentation after manual refinement showing CSF (blue), WM (yellow), GM (green), and blood vessels (red).

Finally, the MR segmentations were visually inspected by a radiology resident (MTHO) with 5 years of experience with stroke imaging. Of each patient, 3 to 4 slices were selected approximately at the level of the Circle of Willis, basal ganglia, lateral ventricles and centrum semiovale, thereby focusing on the supratentorial brain which is most relevant in stroke. Only axial cross sections were selected because in this direction the voxel sizes of MR and CT were approximately the same. We collected in total 87 slices from the 22 patients. The segmentations were then overlaid on the WTA images and manually refined by the same observer with ITK-SNAP. Refinement was required in case vessel voxels were present in the WTA image and to handle potential inaccuracies from the MR to CT registration and FSL segmentation steps. An example WTA image, registered MR and final segmentation at the four different slice levels are shown in Fig. 7.
