# Author Correction: White Matter and Gray Matter Segmentation in 4D Computed Tomography

**DOI:** 10.1038/s41598-018-25729-6

**Published:** 2018-05-15

**Authors:** Rashindra Manniesing, Marcel T. H. Oei, Luuk J. Oostveen, Jaime Melendez, Ewoud J. Smit, Bram Platel, Clara I. Sánchez, Frederick J. A. Meijer, Mathias Prokop, Bram van Ginneken

**Affiliations:** Department of Radiology and Nuclear Medicine, Radboud University Medical Center, Geert Grooteplein 10, 6525 GA Nijmegen, The Netherlands

Correction to: *Scientific Reports* 10.1038/s41598-017-00239-z, published online 09 March 2017

This Article contains an error in Equation 11.

$${\sigma }_{WTA}=\sqrt{\frac{1}{{\sum }_{i=1}^{N}1/{\sigma }_{i}}}$$

should read:

$${\sigma }_{WTA}=\sqrt{\frac{1}{{\sum }_{i=1}^{N}1/{\sigma }_{i}^{2}}}$$

